# High Initial HIV/AIDS-Related Mortality and -Its Predictors among Patients on Antiretroviral Therapy in the Kagera Region of Tanzania: A Five-Year Retrospective Cohort Study

**DOI:** 10.1155/2012/843598

**Published:** 2012-09-02

**Authors:** Kihulya Mageda, Germana Henry Leyna, Elia John Mmbaga

**Affiliations:** ^1^Biharamulo District Council, Department of Health, Biharamulo, Kagera, Tanzania; ^2^Department of Epidemiology and Biostatistics, Muhimbili University of Health and Allied Sciences, P.O. Box 65015, Dar Es Salaam, Tanzania

## Abstract

We examined mortality rates and its predictors from a five years retrospective cohort data of HIV/AIDs patients attending care and treatment clinic in Biharamulo Tanzania. Cox regression analysis was used to identify predictors of mortality. Of the 546 patient records retrieved, the mean age was 37 years with median CD4 count of 156 cells. The mortality rate was 4.32/100 person years at risk with males having three times higher mortality compared to females. Starting Antiretroviral treatment with advanced disease state, body weight below 45 kegs, WHO stage 4 disease, and CD4 cells below 50 were main predictors of mortality. Promoting early voluntary counselling and testing should be given a priority to facilitate timely start of treatment.

## 1. Introduction

By the end of 2010, a total of 34 million people were living with HIV/AIDS worldwide with 2.7 million newly infected in 2010 alone. A total of 1.8 million people died of HIV/AIDS-related death in the same year. Low-and middle-income countries such as those of sub-Saharan Africa are home to 90% of people living with HIV/AIDS globally [1]. The revolution in HIV treatment brought about by combination antiretroviral therapy in 1996 had altered the course of the disease among those living with HIV in high-income countries, but had only reached a fraction of people in low- and middle-income countries [1–3]. Access to antiretroviral therapy in low- and middle-income countries increased from 400 000 in 2003 to 6.65 million in 2010, 47% coverage of people eligible for treatment, resulting in substantial declines in the number of people dying from AIDS-related causes during the past decade. In sub-Saharan Africa 30% fewer death was reported in 2010 as compared to 2004 [1, 4].

In Tanzania, it is estimated that 5.7% of adults aged 15–49 years are infected with the HIV virus. Despite of recent reports of HIV prevalence decline especially among men, HIV/AIDS contributes to the largest proportion of mortality among adult population in the country [5]. As it is the case in most countries, scaling up of antiretroviral treatment is underway in the country. 

Since 2004, the government in collaboration with partners initiated a care and treatment program under the National AIDS Control Program. Care and treatment services have been rapidly implemented and scaled up and became more available at lower-level health facilities [5]. As of the year 2010, a total number of 336,150 HIV-infected persons were enrolled for care and treatment. 

Improving access to ART is expected to reduce mortality among people living with HIV/AID. However in poor settings, this gain may be affected by a number of factors. Studies suggest that survival depends on initial CD4 cells count, body mass index, age, sex, ART adherence and nutrition support [6–9]. Additionally, early diagnosis and early initiation of treatment increase the potential for better outcome. To achieve the expected gain, countries need to address factors that may promote better survival such as initial CD4 count, nutrition support, and adherence to ART. Moreover, mortality following ART initiation could also signal development of viral resistance to ART and poor quality of other health services [9–11]. Therefore it is crucial to track the rate of mortality among HIV/AIDS patients on ART and identify important predictors of mortality to be addressed in the routine care and treatment programmes. Since the start of ART in Tanzania, little is known about the rate and predictors of HIV/AIDS-related mortality among patients who are on ART. We therefore aim at providing such information by analysing data collected from a five-year retrospective chart review of patients on ART in Kagera region where HIV/AIDS was first reported in Tanzania. 

## 2. Materials and Methods

### 2.1. Study Design and Population

This was a retrospective cohort study design of HIV/AIDS patients enrolled in all care and treatment clinics in Biharamulo District between November 2004 and October 2009. Eligibility was also based on being aged 18 years and above and having been on treatment for at least 12 months. 

### 2.2. Study Area

Biharamulo District is one of the 8 districts of Kagera Region, the region where the first AIDS case was reported in the country in 1983. The district has an area of 5,627 square kilometres and a total population of 205,466. The district is located at the North West part of the country where it borders the countries of Rwanda and Burundi with a highway crossing the district to these countries. ART services are provided in five out of 19 health facilities available in the district. 

### 2.3. Sampling and Data Collection

All five health facilities providing care and treatment services in the district were included in this study. At the time of data collection, there were 631 patients enrolled in all the five health facilities during the past five years (selected for this study) who initiated ART. ART initiation for all patients was based on the WHO criteria, and this has remained so to date. Data for all patients from hospital records and Care and Treatment Centre (CTC2) electronic database were examined for eligibility, and those eligible were extracted into a data collection form. Data on sociodemographic and medical related characteristics were extracted. Patients were considered to have failed (achieved an outcome of interest) if they had died of HIV/AIDS-related causes, and the date of death was recorded as failure date. Patients were censored if they had missed three (three months) appointments for drug refill or they were alive at the time of data collection. For those who were alive, the last date of clinic was recorded as the date of censoring. Two data collectors mostly recruited amongst the workers in the respective clinics were trained and performed data collection. 

### 2.4. Ethical Consideration

Ethical approval for this study was obtained from the Muhimbili University of Health and Allied Sciences Ethical Committee. Permission to access ART registers and database was sought from the region and district health authorities and all participating clinics. No name was recorded during data abstraction. 

### 2.5. Data Analyses

Data were entered and analysed using STATA version 11 software. Categorical data were summarized by frequencies and differences between proportions examined using the chi-square test. Continuous variables were summarized using means and respective standard deviations (SDs) or medians as deemed appropriate. Student's *t*-test was used to examine differences between means. 

Median survival time was estimated using Kaplan Meir methods as a point where 50% of the subjects were still alive. Mortality rates were calculated in different categories of predictors, and log-rank test was used to examine differences in mortality rates between these predictors. Cox proportional hazard models were built to estimate the relative hazards of death between levels of confounders. Both univariable and multivariable models were fit. Before building the models, we checked and assured that the proportionality assumption was met. Adjusted hazard ratios (AHRs) with their 95% confidence intervals (CIs) are reported. All the tests were two-tailed, and the type 1 error rate was set at 5%.

## 3. Results

### 3.1. Baseline Characteristics

Out of the 631 patients receiving care and treatment services in the five health facilities in the district, 76 (12.0%) were not eligible (24 were less than 18 years and 52 were on ART for less than 12 months); records of 9 (1.4%) patients were excluded due to missing many variables leaving 546 (86.6%) eligible patients. Data for these patients covered a retrospective period of five years with a total of 47 deaths. Of those eligible, 320 (59%) were female. The overall mean age at ART initiation was 37 years SD ± 10 years with males significantly older than females (males 41, SD ± 11 years versus females 36, SD ± 9 years, *P* values = 0.001). Out of those who participated, 234 (42.9%) were residing in rural areas and 312 (57.1%) in urban areas. In addition 484 (88.6%) of the enrolled patients sought care from a hospital while the rest (*n* = 62, 11.4%) sought care from lower health facilities (dispensaries and health centres). A substantial proportion of men were married while majority of women were separated (*P* = 0.001). Regarding the WHO clinical staging, 376 (68.9%) patients started treatment in stages of the disease (stage 3 and stage 4). More than one-third (201/546, 36.8%,) of patients were in advanced disease (WHO stage 4). The overall median CD4 cell count was 159 cells/mm^3^ (interquarter range (IQR); 65–234). The median CD4 cell count for males was 133 cell/mm^3^ [IQR, (65–222)], and for females it was 178 cells/mm^3^ [(IQR, 75.5–249)], (*P* = 0.033). Relatively lower proportion of men (15.9%) had CD4 count more than 200 cells/mm^3^ as compared to 20.6% of females. The mean weight for this population was 52Kgs with standard deviation (SD) ± 13 kg with males being heavier than females, 54 kg, SD ± 13 and 50 kg SD ± 13, respectively. 

Comparison of patients demographic and medical characteristics by enrolment year is presented in Table 1. The proportions of younger patients aged below 30 years increased over the years from 47.8% to 71.3% (*P* < 0.001). Married individuals formed a large group of patients accessing services in this area during all the years of clinic enrolment included in this study. There was a large proportion of unrecorded medical parameters such as CD4 cells and WHO clinical stage in the clinic enrolment period of November 2005 to October 2006. Other demographic and medical characteristic did not show a clear different pattern over the enrolment period.

### 3.2. Mortality Rates

The overall median survival time after antiretroviral initiation was 24 months (95% CI 21.8–26.36). HIV/AIDS-related mortality rates of the study population by socio-demographic and medical characteristics are depicted in Table 2. The overall mortality rate in this population was found to be 4.32/100 person-years at risk (PYAR) (95% CI: 3.24–5.74) with males (7.84/100 PYAR, 95%  CI:5.51–11.15) having almost three and a half times higher mortality compared to females (2.31/100 PYAR, 95% CI :1.41–3.77). 

Mortality rate in this population was higher during the first five months following the start of ART and thereafter decreasing, remaining below 5.0/100 PYAR after six months of treatment. Being single or coming from urban area was associated with higher mortality rates in this population. 

Body weight below 45 kg and WHO stage 4 were associated with higher rates of mortality in the crude bivariate analysis. 

### 3.3. Predictors of Mortality

Male patients on ART had five times higher rate of death as compared to female patients in this population. Place of residence was found to be a significant predictor of mortality in this population where patients from urban areas had two times higher death hazard as compared to those from rural areas (HR = 2.16(95% CI :1.20-3.90). Starting ART with lower body weight of less than 45 kgs was associated with a 4.99 (95% CI :1.91–7.29) higher death hazard as compared to those starting ART with body weight above 55 kg. Death hazard rate was five times higher among patients starting ART with CD4 count less than 50 cells/mm^3^ while those starting treatment with CD4 cells between 50–199 cells/mm^3^ had two times higher hazard as compared to those starting ART with more than 200 cells/mm^3^. Disease stage based on WHO classification was a predictor of mortality among the study population. WHO stage 4 at the start of ART was associated with four times higher death rate as compared to WHO stage 1–3 combined (Table 3).

## 4. Discussion

The use of antiretroviral therapy has been associated with prolonged survival among people living with HIV/AIDS in the world [1, 12]. The median survival time after ART initiation during the study period was found to be 24 months in this population. This was comparable to what was reported in a study done in Ethiopia by Andinet and Sebastian [13]. However, median survival time in this population was lower than that reported in other studies in the country [14] and that done in Uganda [15]. On the other hand, patients in this study survived longer than those reported in studies conducted in South Africa [16, 17]. These variations indicate that although ART increases survival, it is not the only determinant of survival among people living with HIV/AIDS but could depend on the characteristics of the patients, adherence, and quality of service provision [6, 18]. 

Mortality in this study was found to be 4.3/100 person-years at risk with more death occurring during the first five months following ART initiation. These findings are similar to what has been reported elsewhere [9, 10, 19]. High death rate in the beginning of ART use has been associated with immune reconstitution inflammatory syndrome especially for patients staring treatment with advanced diseases (WHO stage 4) [10, 19–21]. A study in Ethiopia established that the high mortality during the first months of treatment was strongly associated with advanced clinical stage and weight loss [10]. Additionally, studies in Tanzania and South Africa observed that there were more deaths occurring within three months after ART start and were strongly associated with anaemia, thrombocytopenia, and severe malnutrition [14, 16, 17]. 

A large proportion (68.9%) of patients in this population was found to start ART with disease stage 3/4 with more than one-third (36.8%) starting with advanced WHO stage 4 disease. This proportion was significantly larger as compared to the national average of 35% for WHO stage 3/4 disease [1]. However, the median CD4 cells of 156 cells/mm^3^ at the start of ART were in the range of what has recently been reported in East Africa (154 cells/mm^3^) [22] which was also comparable to contemporaneous estimates of 187 cells/mm^3^ in the United States, 159 cells/mm^3^ in Brazil, and 157 cells/mm^3^ in China [23]. The large proportion of advanced disease patients in this study could partly be explained by the service provision nature of distantly located districts and regions in country. Only 5 out of 19 health facilities in this region offered ART services, and this could somewhat limit easy access especially for those residing far from the facilities. Most people from larger cities such as Dar Es Salaam where many facilities can offer HIV testing and ART could timely access health education, HIV testing, and ART as compared to region with limited resources.

This study also showed males to have higher mortality rate than females. This finding was similar to other studies in Africa which showed mortality rate to be higher in males than in females [7, 19, 24]. Late reporting of men to care and treatment clinics and poor adherence to treatment are among the reasons identified by various studies [23–25]. Studies suggested that female patients tend to know their HIV status and start antiretroviral therapy early with better CD4 cell count compared to males. This has been explained to be due to the linkage between prevention of mother-to-child transmission (PMTCT) and care and treatment clinics [24]. Most patients especially men would go for HIV testing after experiencing HIV/AIDS-related symptoms. As found in this study, a study involving three east Africa, countries has indicated that males start ART with advanced disease as compared to female [22]. The study also indicated that the advanced disease profile of men as compared to women at the starting ART could not fully be explained by the public health services giving women a PMTCT testing advantage. This was because men were 50% more likely to assess ART with advanced disease as compared to women without any history of PMTCT. Further studies were suggested to examine causal reason for late reporting among men [22]. 

Patients residing in urban areas had higher death rates as compared to those from rural communities. Traditionally, rural communities are more supportive, extended with social cohesion than urban communities. People living with HIV/AIDS could benefit from these supports which may include drug adherence support, nutrition support, and other sick role benefit that may be instrumental in survival. Moreover, relatively fewer number of HIV infected patients in rural areas could make it easy for the health system to support them adequately and effectively with the help of community HIV/AIDS support staff. 

Lower body weight (cachexia or wasting syndrome) and lower CD4 count are proxy measures of advanced disease stage. Patients stating ART with advanced disease stage as defined by WHO stage 4 are associated with increased risk of immune reconstruction syndrome and death. In this study we found that patients with advanced disease WHO stage 4 mostly characterised by lower body weight (weight below 45 kg) and lower CD4 count (less than 50 cells/mm^3^) were associated with higher rate of death. Low body weight has also been associated with malnutrition hence poor immunity among HIV infected-individuals. These findings were consistent with that of a study done in Ethiopia [13], where patients who were given antiretroviral therapy with low body weight had higher mortality. Another study in Uganda reported that CD4 count above 200 cells/mm^3^ was associated with better survival among HIV-infected patients [15]. These findings underpin the importance of promoting voluntary and HIV testing to facilitate early disease diagnosis and early ART start hence relatively low associated mortality.

Many countries including Tanzania have started to provide nutritional support to people living with HIV who are on ART. This is now part of the HIV care and treatment policy but its implementation has not been effective as most clinics are not offering this service. Lack of nutritional support and late HIV diagnosis have been a drawback to the anticipated positive effects of ART scale-up in poor countries.

The findings of this study should be interpreted in light of the following limitations; our analysis was based on secondary data that were not collected for research purposes. Important predictors of survival such as drug adherence, economic status, and drug resistance were missing. 

## 5. Conclusion

HIV-related mortality especially among men was high, and mean survival time was relatively low in this population. Majority of patients started ART at a late disease stage characterised by wasting syndrome, lower CD4 cells, and these were associated with higher mortality. Scaling up voluntaray and counselling and testing in the general population as well as through male involvement in reproductive health services would promote early diagnosis and early ART initiation. Further steps to implement the proposed policy on nutrition support among patients on ART would prove beneficial in reducing HIV-related mortality. 

## Figures and Tables

**Table 1:** Distribution of sociodemographic and medical characteristics by enrolment period among patients attending care and treatment clinics in Biharamulo District, Tanzania.

Variable	Year of enrolment					Total
	Nov. 2004–Oct. 2005	Nov. 2005–Oct. 2006	Nov. 2006–Oct. 2007	Nov. 2007–Oct. 2008	Nov. 2008–Oct. 2009	
Sex						
Male	4 (17.4)	45 (44.5)	53 (37.6)	60 (41.4)	64 (47.0)	226
Female	19 (82.6)	56 (55.5)	88 (62.4)	85 (58.6)	72 (52.9)	320

Total	23 (4.2)	101 (18.5)	141 (44.1)	145 (26.7)	136 (24.9)	546

Age						
<30	11 (47.8)	54 (53.5)	77 (54.6)	91 (62.7)	97 (71.3)	330
40+	12 (52.2)	47 (46.5)	64 (45.4)	54 (37.3)	39 (28.7)	216

Total	23 (4.2)	101 (18.5)	141 (44.1)	145 (26.7)	136 (24.9)	546

Residency						
Rural	10 (43.5)	54 (53.5)	81 (57.4)	88 (60.7)	79 (58.1)	312
Urban	13 (56.5)	47 (46.5)	60 (42.5)	57 (39.3)	57 (41.9)	234

Total	23 (4.2)	101 (18.5)	141 (44.1)	145 (26.7)	136 (24.9)	546

Type of clinic						
Hospital	22 (95.6)	98 (97.0)	126 (89.3)	132 (91.0)	106 (77.9)	484
Lower facility	1 (4.4)	3 (3.0)	15 (10.4)	13 (9.0)	30 (22.1)	62

Total	23 (4.2)	101 (18.5)	141 (44.1)	145 (26.7)	136 (24.9)	546

Marital status						
Never married	3 (13.0)	10 (9.9)	16 (11.3)	25 (17.2)	20 (15.3)	74
Married	6 (26.2)	28 (27.7)	64 (45.4)	56 (38.6)	74 (56.5)	228
Separated	7 (30.4)	20 (19.8)	44 (31.2)	48 (33.1)	33 (25.2)	152
Not recorded	7 (30.4)	43 (42.6)	17 (12.1)	16 (11.0)	4 (3.0)	87

Total	23 (4.2)	101 (18.7)	141 (26.1)	145 (26.8)	131 (24.2)	541

Clinical stage						
1 to 3	11 (47.8)	15 (14.9)	49 (34.7)	68 (46.9)	85 (62.9)	228
4	10 (43.5)	31 (30.7)	57 (40.5)	67 (46.2)	46 (34.1)	211
Not recorded	2 (8.7)	55 (54.4)	35 (24.8)	10 (6.9)	4 (3.0)	106

Total	23 (4.2)	101 (18.5)	141 (25.9)	145 (26.6)	135 (24.8)	545

CD4 count						
<50	6 (26.1)	4 (4.0)	15 (10.6)	20 (14.7)	17 (12.5)	62
50 to 199	9 (39.1)	5 (4.9)	35 (24.8)	27 (18.6)	40 (29.4)	116
200+	1 (4.3)	5 (4.9)	33 (23.4)	37 (25.5)	32 (23.5)	108
No baseline	7 (30.5)	87 (86.2)	58 (41.1)	61 (42.2)	47 (34.5)	260

Total	23 (4.2)	101 (18.5)	141 (44.1)	145 (26.7)	136 (24.9)	546

Weight						
<45	9 (39.2)	17 (16.8)	32 (22.7)	33 (22.8)	35 (25.7)	126
45 to 54	8 (34.8)	23 (22.8)	67 (47.5)	48 (33.1)	56 (41.2)	202
55+	5 (21.7)	33 (32.7)	37 (26.2)	60 (41.4)	39 (28.7)	174
Not recorded	1 (4.3)	28 (27.7)	5 (3.6)	4 (2.7)	6 (4.4)	44

Total	23 (4.2)	101 (18.5)	141 (44.1)	145 (26.7)	136 (24.9)	546

**Table 2:** Mortality rates by sociodemographic and medical characteristics of patients in care and treatment centre in Biharamulo District.

Variable	Number of people (*n*)	Death	PYAR	MR (Per 100 PYAR)	MRR (95% CI)	*P* Value^‡^
Overall	546	47	1089	4.31	3.24–5.74	
Sex						
Male	226	31	396	7.83	5.51–11.14	0.001
Female	320	16	693	2.30	1.41–3.76	
Age at ART start						
18–24	137	3	110	2.72	1.12–6.52	0.201
25–34	163	18	386	4.66	2.13–7.90	
35–44	188	22	497	4.43	1.80–7.58	
>44	58	4	96	4.16	1.32–9.12	
Marital Status						
Never married	77	8	152	5.27	2.64–10.54	0.050
Marriage	228	17	476	3.57	2.22–5.75	
Separated/divorced	155	11	326	3.37	1.87–6.09	
Not recorded	86	10	135	7.67	4.13–14.26	
Residence						
Rural	234	18	601	2.98	1.88–4.73	0.018
Urban	312	29	488	5.96	4.14–8.58	
Type of clinic						
Hospital	484	45	967	4.65	3.47–6.23	0.131
Lower facility	62	2	122	1.65	0.41–6.56	
Body weight (kg)						
<45	125	25	247	10.1	4.60–19.58	0.021
45–54	203	9	418	2.15	1.12–4.14	
55+	174	12	379	3.16	1.80–5.57	
Not recorded	44	1	45	2.22	0.62–8.73	
Pre-ART CD4 count						
<50	76	10	106	9.41	5.06–17.49	0.030
50–199	260	8	228	3.51	1.75–7.01	
200+	102	26	538	4.84	3.29–7.10	
No baseline CD4	108	3	217	1.38	0.45–4.28	
WHO Pre-ART						
1–3	239	8	433	1.84	0.92–3.68	0.001
4	201	30	412	7.28	5.09–10.41	

PYAR: person-years at risk; MR: mortality rate; CI: confidence interval, ^‡^ *P* value from log-rank test.

**Table 3:** Unadjusted and adjusted hazard ratios of determinants of mortality among patients receiving ART in Biharamulo District.

Variable	Categories	*N*	Mortality rate	Unadjusted HR^*α*^	Adjusted HR	*P* Value
Sex	Male	226	7.83	3.19 (1.74–5.84)	4.71 (2.00–11.05)	0.001
	Female	320	2.30	1	1	
Age group (years)	18–24	137	2.72	1	1	0.461
	25–34	163	4.66	2.12 (0.91–7.11)	1.82 (0.81–9.87)	
	35–44	188	4.43	2.00 (0.89–10.17)	1.76 (0.88–13.28)	
	>44	58	4.16	1.96 (0.91–12.65)	1.98 (0.74–15.77)	
Marital status	Never married	77	5.25	1.46 (0.63–3.39)	1.70 (0.65–4.87)	0.302
	Married	228	3.57	1	1	
	Separated/divorced	155	3.37	0.95 (0.45–2.05)	1.01 (0.52–4.56)	
Type of clinic	Hospital	484	4.65	1	1	0.159
	Lower facility	62	1.65	0.57 (0.01–1.12)	0.64 (0.12–2.16)	
Residence	Rural	234	2.98	1	1	0.004
	Urban	312	5.96	2.05 (1.14–3.70)	2.16 (1.20–3.90)	
Body weight	<45	125	7.30	3.44 (1.62–7.29)	4.99 (1.91–7.29)	0.023
	45–54	203	2.15	0.30 (0.13–0.97)	0.23 (0.10–1.01)	
	55+	174	3.16	1	1	
CD4 Count	<50	76	9.41	6.70 (2.13–10.45)	5.23 (2.09–10.69)	0.002
	50–199	260	3.51	2.50 (1.18–12.11)	2.12 (1.11–13.26)	
	200+	102	1.38	1	1	
WHO stage	1–3	239	1.84	1	1	0.001
	4	201	7.28	4.16 (1.91–9.09)	4.19 (1.92–9.16)	

^*α*^HR: hazard ratio.
